# Foreign Psychological Literature

**Published:** 1860-10-01

**Authors:** 


### FOREIGN PSYCHOLOGICAL LITERATURE.

OUR retrospect of Foreign Psychological Literature will embrace the following subjects:—

1. On Hybridity in Man, and the Plurality of Human Species.
2. On Private Asylums for the Insane.
3. On the Management of the Insane in Belgium.
4. Patronal Asylums.
5. On the Curability and Cost of Insanity.
6. On Pellagra in Italy, and more especially in the Lunatic Asylums of that country.
7. On Microcephalus and the characteristics of the Human Race.

### 1. *On Hybridity in Man, and the Plurality of Human Species.* By Dr. PAUL BROCA.

THE following are the results derived by Dr. Paul Broca from a series of valuable and highly interesting researches on human hybridity:—

1. Certain human crosses are perfectly eugenetic.

2. Other crosses give results which appear to be notably inferior to those of eugenetic hybridity.

3. Mongrels, *de premier sang*, issues of a cross between the Germanic (Anglo-Saxon) race and the African negro, appear to be inferior both in fecundity and longevity to individuals of a pure race.

4. It is at least doubtful whether these mongrels, in alliances among themselves, are capable of perpetuating their race indefinitely, and whether they are less fertile in their direct alliances than in their return crosses with the two mother races, as is observed in paragenetic hybridity.

5. The crosses of the Germanic (Anglo-Saxon) race with the Melanesian races (Australians and Tasmanians) are little fertile.

6. Mongrels, the issue of this cross, are too rare to enable us to form an opinion upon their viability and fecundity.

7. Many of the degrees of hybridity which have been demonstrated in the crosses of animals of different species appear to be reproduced in the divers crosses of men of different races.

8. The lowest degree of human hybridity, that in which the homœogenesis is so feeble as to render doubtful the fecundity of the first cross, is manifested when the greatest disparity in the cross occurs, to wit, between one of the most elevated races and the two lowest races of humanity.

Dr. Broca then proceeds:—

The many numerous and controverted questions which we have had to discuss before arriving at the end of our work have more than once broken the chain of the argument. It will therefore not be without profit to attempt to bring together its divers parts.

Zoologists have recognised in *each* of the natural groups which constitute genera *several* distinct types which they designate by the name of species.*

The human group evidently constitutes one genus; if it contain but a single species it will be an unique exception in the creation. It is natural then to think that this genus, like all others, is composed of several species.

In a great number of genera, the species differ much less from one another than do certain of the human races. A naturalist who, without troubling himself as to the question of origin, would apply purely and simply to the human genus the general principles of zootaxy, would then be led to divide this genus into several species. It would only become necessary to renounce this view if observation demonstrated that all the differences of the human races have been the result of modifications impressed on the organization of man by the influence of surrounding circumstances.

Monogenists are called upon for this demonstration at the outset. They have not yet been able to arrive at it. Observation has, on the contrary, demonstrated that if the organization of man does sometimes undergo in the long run, and in the course of generations, some modifications under the influence of exterior conditions, these modifications, relatively very slight, have no relation to the typical differences of the human races. Man transplanted to a new climate, and submitted to a new kind of life, preserves and transmits to his posterity the essential characteristics of his race, and his descendants acquire no more than himself the characteristics of the indigenous race or races. *Cœlum non* corpus *mutant qui trans mare currunt.*

Monogenists object that the era of far distant colonies is too recent; that the observations tending to establish the permanence of the human types date scarcely three or four centuries; that this lapse of time is insufficient for the operation of the transformation of races, and that this transformation was produced and gradually aggravated during the long course of ages which has elapsed, since the creation of man, according to some, since the deluge only, according to others.

But the study of Egyptian pictures has shown on the one hand that the principal types of the human genus already existed such as they are at the present day 2500 years at least before the Christian era; on the other hand, that the Jewish race, dispersed for more than eighteen centuries under the most various climates, is the same to-day in every country as it was in Egypt at the epoch of the Pharaohs.

The period of *positive* observation dates then from more than forty *centuries*, and not merely three or four.†

* Some genera, which in the existing fauna only include *a single* species, are represented in anterior fauna by a certain number of species now extinct, and evidently different from the sole remaining species.

† There exists, at the present day in Southern Africa, and as far as the Sahara, a race of men with white hair, which some have wished to identify as the descendants of the Vandals. It is certain that no white race has established itself in that region since Genseric; that is to say, within fourteen centuries. It results from this, therefore, that fourteen centuries of sojourn on the African continent do not suffice to blacken the hair of white men. But Dumoulin, basing his statements on

No longer hoping to prove directly that the distinctive characters of the human races are born of the transformations of an unique primitive type, the monogenists have sought for indirect proofs. They have thought the discovery made in the fact, or rather in the assertion, that there is, if not a constant relation, at least a certain connexion between the characters of the human races and the centres in which they are found.

But, on closer examination, it could not fail to be acknowledged that this assertion is without foundation. Taking one by one the principal ethnological characteristics, and considering their distribution over the surface of the globe, we have shown to demonstration that there is no relation between these various characteristics and climateric, hygienic, or other conditions.

The monogenists have then had recourse to a still more indirect argument. They have asserted that there is, throughout the human race, a common fund of ideas, of belief, of knowledge, and of language attesting the common origin of all races. It might be objected, in the first instance, that this argument was entirely without value, considering that even very indirect communications between two peoples of different origin might be sufficient to convey from one to the other words, customs, and ideas. But we find, upon a more attentive study of the question, that certain nations have absolutely no notion of God, or of the soul; that their languages have absolutely no point of contact with ours, that they are totally unsocial, and that they differ from the Caucasian nations in their intellectual and moral even more than in their physical characteristics.

It did not even appear necessary to insist on the difficulty, or rather on the geographical impossibility, of the dispersion of so many races proceeding from a common origin, nor to remark that, before the distant and almost recent migrations of Europeans, each natural group of the human races occupied a region on our planet characterized by a special fauna; that no American animal was to be found in Australia, nor in the ancient continent, and that where we discovered men of a new type, we only met with animals belonging to species, often even to genera, and sometimes to zoological orders, without analogues in the other regions of the globe.

And while it was so easy to conceive that there had been several centres of creation for men as well as for other beings; while this doctrine, conforming itself to all the data of the natural sciences, caused all geographical obstacles to disappear; while it explained so well at once the analogies and the differences of the human types, and the distribution of each group of races; while, in a word, it rendered such an exact account of all known facts, the opposite doctrine struggled in a circle of contradictory suppositions, superposed hypotheses, of theories the text of Procopius, had already demonstrated that the white race of Southern Africa had nothing in common with the Vandals; and I have recently discovered, in *Le Périple de la Mediterannée* of Scylax, a work anterior to Alexander the Great, a passage in which mention is made of a tribe of *white* Libyans, who occupied the littoral of little *Syrte*, not far from Mount Auress, where reside at the present day one of the chief tribes of white Kabyles. *Vide Bulletins de la Soc. d'Anthropologie, Séance du* 16 *Fev.* 1860.

built upon a small number of facts soon overthrown by others, of imaginary influences belied by observation, of prehistoric romances annihilated by the discovery of old monuments of history, of lame explanations demolished by physiology, of nebulous sophisms repelled by logic—the whole in order to show, not that all races descend from a common origin, but that the thing, strictly speaking, may not be quite impossible!

Where have the monogenists gathered courage and perseverance to impose such continual sacrifices upon their reason, and to resist at once the testimony of observation, of science, and of history? When their system is analysed, we meet at every turn two fundamental axioms, which are to them as articles of faith, and the evidence of which appears to them sufficient to carry them over all difficulties. These two axioms stand as premises of a syllogism, in appearance irresistible.

1. *All animals capable of engendering an eugenetic posterity are of the same species.*

2. *All human crosses are eugenetic, therefore all men are of the same species.*

Believing themselves sure of the two premises of this syllogism, monogenists have considered their doctrine as rigorously and definitively established; thence they have defended it with that unlimited confidence which results from absolute conviction. Assailed by pressing objections, obliged to yield incessantly, and unable to make a step in advance without being compelled as speedily to retreat, they have always felt their strength renewed on returning to the cover of their syllogism, like Antæus on touching the earth. So long as this refuge shall remain, they will continue the struggle, if not with advantage, still with the ardour of faith; for if faith no longer removes mountains, it still leaves the belief that they are removed.

But are these fundamental propositions, these admitted axioms—are they the expression of the truth? This triumphant syllogism, of which they are the premises, does it still stand on its legs? Is it true that only animals of the same species can produce a fruitful posterity? Is it true that all human crosses are eugenetic?

It would suffice if the first of these questions were answered in the negative to destroy the monogenists' syllogism, and to deprive their system of all scientific support; it would again become what it was before being brought into contact with science, that is to say, a belief more or less respectable, based upon sentiment or dogma. But if the second question should also in its turn receive a negative reply, if it were demonstrated that all human crosses are not eugenetic, then, not merely the syllogism but the entire doctrine of the monogenists must crumble away. This doctrine would then be not merely extra-scientific, it would be anti-scientific; for it is sufficiently certain that two groups of animals different enough to be incapable of fusion by generation, do not belong to the same species. This is a truth uncontested as it is incontestable.

We have thus been led to examine successively the two fundamental propositions on which is based the Unitarian doctrine, and in order to this we have had to undertake two series of researches.

We have studied, in the first place, the results of certain crosses between animals of unquestionably different species, such as dogs and wolves, sheep and goats, camels and dromedaries, hares and rabbits, &c., and we have demonstrated that *these crosses produce eugenetic mongrels, that is to say, perfectly and indefinitely fruitful between themselves.*

It is therefore not true that all animals capable of producing eugenetic posterity are of the same species, and even should all human crosses be eugenetic, as generally believed, one cannot thence conclude anything relative to the question of the unity of the human species.

The monogenists are thus henceforward deprived of their principal and only scientific argument.

But it was required to know further, whether the vulgar axiom that all human crosses are eugenetic was a demonstrated truth, or an hypothesis lightly accepted without verification or control. Such has been the object of our second series of researches.

We noticed in the first place that monogenists, treating this axiom as self-evident, had not even sought to prove its truth, so that in strictness we might have been at liberty to discard it as not.

When we sought to establish, contrary to the opinion of many modern authors, that there are really eugenetic crosses amongst the human races, we found in the scientific writings on the subject nothing but assertion without proof, and we believe that our studies respecting the crossed populations of France have in this aspect the merit of novelty. We may be mistaken respecting the value of our demonstration, but we venture to assert that it is the first which has been attempted.

After having shown it to be, if not altogether certain, at least extremely probable, that *some* human crosses are eugenetic, it became our duty to ask if *all* human crosses possessed the same attribute.

Upon examination of this question, it followed from the documents we were able to collect, that certain human crosses appeared to give results notably inferior to those which constitute eugenetic hybridity in the case of animals. The sum total of known facts permits us to consider as very probable that certain of the human races taken two and two are less homœogenetic than are, for example, the species of dog and wolf. If we deem it our duty to make this assertion with some reserve, if we allow some appearance of doubt to rest upon this conclusion, it is because we should not feel justified in admitting without numerous verifications a fact which would definitively and for ever demonstrate the plurality of the human species, a fact in presence of which all others would become unimportant, and which would render all further discussion superfluous, a fact, indeed, the political and social consequences of which must be immense.

We feel we cannot too strongly insist upon the importance of drawing the attention of observers to this subject. But, whatever may be the result of ulterior researches relative to human hybridity, it is well proved that animals of different species can engender eugenetic mongrels, and that therefore we cannot draw from the fecundity of the most dissimilar of human crosses any physiological argument in favour of the unity of the human species, even were this fecundity as certain as it is doubtful.

The great problem which we have discussed in this essay is one of those which have deeply interested mankind, one of those which it is most difficult to study with a mind free from all extra-scientific bias. Hitherto it has been mixed up with religion and politics. This was almost inevitable, but science must contrive to hold herself aloof from all that does not belong to her. There is no belief so respectable, there is no interest so legitimate, that it ought not to accommodate itself to the progress of human knowledge, and bow before the truth when the truth is demonstrated. It is therefore always unadvisable to interpose theological arguments into debates of this nature, and to stigmatize in the name of religion such and such scientific opinion, because if that opinion should sooner or later come to be established, one is open to the reproach of having uselessly compromised religion. The unlucky intervention of theologians in the questions of astronomy (rotation of the earth), of physiology (pre-existence of germs), of medicine (possessions), &c., has made more unbelievers than all the writings of philosophers. Why should men thus be put to their election between science and faith? And whilst so many celebrated examples have compelled theologians to acknowledge that revelation is not applicable to questions of science, why persist still in throwing the Bible under the wheels of progress? Some sincere Christians have already understood that the time has come to prepare a reconciliation between the doctrine of polygenists and the sacred text. They are disposed to admit that the narration of Moses does not apply to all the human race, but solely to the *Adamites*, the race from whence sprung the people of God; that there might be upon the earth other men, respecting whom the sacred writer did not concern himself; that it is nowhere stated that the sons of Adam contracted incestuous unions with their own sisters; that Cain, banished towards the East after the murder of his brother, was marked with a sign, "in order that whosoever found him might not kill him;" that besides the race of the children of God, there was the race of the children of men; that the origin of the children of men is not specified; that nothing authorizes us to consider them as the children of Adam; that these two races undoubtedly differed in their physical characters, because their union produced offspring denominated by the name of giants, "as if to indicate the physical and moral energy of the crossed races;" that in fact these diverse antediluvian races might have been able to survive the deluge in the persons of Noah's three daughters-in-law.* We unite here the reflections of several authors; one of them, the Rev. Pye Smith, concludes by saying with satisfaction, that if, contrary to actual opinion, the multiplicity of human species should come to be demon-

* J. Pye Smith, *Relations between the Holy Scripture and Geology*, 3rd ed., pp. 398—400. Passage textually reproduced by Morton in *A Letter to Rev. John Bachmann on Hybridity*, Charleston, 1850, 8vo, p. 15; Carpenter, article *Varieties of Mankind* in Todd's *Cyclopædia of Anatomy and Physiology*, vol. iv., p. 1317, Lond., 1852, 8vo; Eusebe de Salles, *Histoire général des races humaines*, Paris, 1849, 12mo, p. 328.

strated, a thing he thought little probable, the authority of the Bible would remain intact, and that "the highest interests of man would not suffer by it." This is one mode of reconciliation already prepared in anticipation of the ulterior developments of science. More recently, a fervent Catholic, a physician, who during long voyages has attentively studied the human races, M. Sagot, has put forth an hypothesis which we think is quite new, and which will permit us still better than the preceding to reconcile the biblical narrative with anthropological science. After having pointed out with much force that the physical, intellectual, and moral characteristics of the human races establish the existence of profound differences between them, that these differences are quite indelible, that all the influences to which they have been attributed are absurd and imaginary, and that natural causes could not have produced such a diversity from primitive uniformity, M. Sagot supposes that the division of the human species into perfectly distinct races was, like their dispersion and methodical distribution over the surface of the globe, the result of a miraculous intervention of Providence. He thinks that this great fact was brought about at the period of the confusion of tongues, that is to say, after the rash enterprise of the Tower of Babel, and that God in dispersing the families gave to each a peculiar organization and aptitudes suited to the various climates he assigned to them.* That the differences of the human races have been the consequence of distinct creations, or of miraculous transformations equivalent to new creations, this comes to the same thing, so far as concerns the doctrine of the polygenists. Their object is not to give themselves to theological discussions; they have only set foot on this ground because they were drawn there, and they will be enchanted to learn that their doctrine may henceforward develop itself without grieving any one.

The intervention of political and social considerations has not been less troublesome to the cause of anthropology than that of the religious element. When generous philanthropists called with indefatigable constancy for liberty to the black man, the partisans of the ancient order of things, menaced in their dearest interests, were glad to say that Negroes were not men, but only domestic animals more intelligent and productive than others. At this epoch the scientific question gave way to a question of sentiment, and whoever joined in vows for the abolition of slavery, believed himself obliged to admit that Negroes were Caucasians blackened and frizzled by the sun. Now that the two greatest civilized nations, France and England, have finally emancipated slaves, science may reclaim its rights without troubling itself about the sophisms of slaveholders.

Many good people imagine, however, that the time for speaking with all freedom has not yet arrived, because the battle of emancipation is far from being terminated in the United States of America, and that it is necessary to avoid furnishing arguments to the supporters of slavery. But, is it true that the polygenist doctrine, which scarcely

* P. Sagot—*Opinion générale sur l'Origine et la Nature des Races humaines; Conciliation des Diversités indélébiles des Races avec l'Unité historique du Genre humain.* Paris, 1860; 8vo, 80 pages. (Arthur Bertrand).

dates a century back, can be in any degree responsible for a state of things which has existed from time immemorial, and which was developed and perpetuated during a long course of ages under the shadow of the doctrine, so long uncontested, of the monogenists? And does any one believe that slaveholders are at all embarrassed to find arguments in the Bible? The Rev. John Bachmann, a furious monogenist of South Carolina, has acquired great popularity in the southern States by demonstrating, with considerable unction, that slavery is a divine institution.* It is not from the writings of polygenists, but from the Bible that the representatives of the Slave States have drawn their arguments; and Mr. Bachman tells us that the abolitionists of Congress stood silent before this irrefragable authority. Let us then cease to think that there is the slightest connexion between the scientific and the political question. The difference of origin in no way implies the subordination of races. It implies, on the contrary, that each race of men has had its rise in a predetermined region; that it has been, as it were, the crown to the fauna of that region. And if it be permitted to assign an intention to nature, we might well believe that she wished to grant a distinct appanage to each, because, notwithstanding all that has been said about the cosmopolitanism of man, the inviolability of the domain of certain races is insured to them by their climate.

Let us compare, now, this mode of viewing the question and that of the monogenists, and let us ask which of the two is best suited to please the partisans of slavery? If all men descend from a single couple—if the inequality of races is the result of a curse more or less deserved—or, again, if these have degraded themselves, and have allowed the primitive spark of intelligence to become extinct, whilst those have kept intact the precious gifts of the Creator—in other terms, if there are races under a blessing, as others are under a curse,—races which have responded to the aspirations of nature, and races which have contemned them—then the Rev. John Bachmann is right in saying that slavery exists by divine right; it is a providential punishment, and it is just, up to a certain point, that the races which have degraded them-

* It may be permitted to us to reproduce a few passages from a dissertation by this pious slaveholder. We extract them from the *Charleston Medical Journal and Review*, Sept., 1854, vol. ix., pp. 657—659. "All the races of men, Negroes included, are of the same species and the same origin. The Negro is a striking and now permanent variety, similar to numerous varieties of domestic animals . . . The Negro will remain what he is unless his form be changed by a cross, the mere idea of which is revolting to us. His intelligence, although too much defamed, is greatly inferior to that of the Caucasians, and he is therefore, from all that we know, incapable of self-government. He has been placed under our *protection*. The defence of slavery is contained in Holy Writ. The Bible teaches the rights and the duties of masters, that slaves be ruled with justice and kindness, and it enjoins obedience to the slave . . . . The Bible furnishes us the best arms we can make use of. It shows us that the ancient Israelites possessed slaves. It determines the duties of masters and slaves; and St. Paul wrote an Epistle to Philemon praying him to take back a runaway slave. Our representatives in Congress have made use of arguments drawn from Holy Scripture, and their adversaries have not dared to say that the historical parts of the Bible (and all that relates to slavery is historical) are false and uninspired." The Rev. John Bachmann adds, a little further on, "We can effectually defend our institutions from the WORD OF GOD."

selves should be placed under the *protection* of others—borrowing an ingenious euphemism from the language of slaveholders. But, if the Ethiopian is King of Soudan, by the same title that the Caucasian is King of Europe, by what right shall this one impose his laws on that, excluding the right of force? In the first case, slavery presents itself with a certain appearance of legitimacy which may render it excusable in the eyes of some theorists; in the second, it is a deed of pure violence against which all protest who do not profit by it.

From another point of view it may also be said that the doctrine of the polygenist assigns to the inferior races of humanity a more honourable position than the opposite. To be inferior to another, be it in intelligence, in vigour, or in beauty, is not a humiliating condition. One must blush, however, for having undergone a physical or moral degradation, at having descended in the scale of beings, and at having lost rank in creation.—(*Journal de la Physiologie de l'Homme et des Animaux*, *Avril*, 1860).

---

2. *On Private Asylums for the Insane.* By Dr. Ed. Jarvis, Dorchester, Massachusetts, U.S.

The subsequent observations of Dr. Jarvis form portion of a paper read by him before the last meeting of the Association of Medical Superintendents of American Institutions for the Insane. The opinions of this distinguished American alienist physician possess considerable interest for the English reader:

"In this country (America) there is a very common notion, that the mere personal influence of a physician, or of any other individual, in a private house, is insufficient for the control and the management of the insane; and this has made our people look to institutions endowed with large facilities and power, with authority and means of securing the unwilling, coercing the refractory, and of amusing and occupying all, in their various moods of excitement and depression. These advantages are to be found only in establishments which are beyond the means of individuals, and not by any probability, perhaps not by any possibility, within their reach. Hence our people look to the body politic, the great aggregation of the common wealth and power, to unite and produce such an establishment as seems to be needed.

"It is supposed by some that the insane in the United States are more maniacal, wilful, and excitable, and consequently need a firmer government, and often more restraint, than those of Great Britain. This, whether true or not, tends to corroborate the first opinion, and induces the more general desire not only to build hospitals with the means at least for keeping such patients, but also to place their own friends in such places as seem to them to be prepared to meet all the emergencies of insanity. Hence all classes here have sent their friends to the public asylums, and having once tried the experiment, they have found good reason to repeat it on any future occasion. A second consequence is, that there has been but very little call, and very little provision made and offered, for the private treatment of the insane.

"The question now arises, What are the peculiar advantages of public, and what of private asylums for the insane? The great majority of cases are better provided for in the public than in private institutions. The wild, the violent, are more secure, with the strong walls, and doors, the guarded windows, and other means of preventing injury or escape. The large corps of attendants, and constant presence of officers, give more moral and personal authority to restraint. If there be need of force, mechanical or other means of restraint, if outbreaks of violence are to be overcome, and injury prevented, or food or medicine to be forcibly administered, the means, both material and personal, for effecting these purposes, can be furnished better in a public establishment than in a private house. The suicidal patients, who seem to have an almost superhuman skill and perseverance in baffling the vigilance of guardians, need all the architectural securities of the buildings, yards, &c., of the best institutions that are designed and built for the insane, and all the combined watchfulness of a trained corps of attendants, to save them from self-destruction. The wilful, perverse, opinionated patients, in whom self-esteem is, by nature or by disease, largely developed, bear opposition to their plans, contradiction to their opinions, and interference and restraint upon their conduct from men in authority, clothed with official power; and when it seems to be the law of the institution which many others recognise and obey, better than when it comes from an individual who has nothing but his personal and professional character to rest upon, and no other law than his own private judgment, however well founded or wisely established.

"But there are some classes of patients, who, from their peculiarity of feelings, or temperament, or disease, would do as well, and some would do better, in a private than in a public asylum. One class inseparably associate the idea and the name of an asylum or of a hospital with disgrace, which they think attaches ever afterwards to those who have resided in them as patients. They think this will be a mark upon them, to lower their claims on the world for respect and confidence in social and commercial life, and lessen their influence in society and business circles. It is not many years since people generally considered insanity as a stigma upon character, and depreciation of mental power, even after perfect recovery, and that however fully the mental health may be restored, nothing would or could restore the broken reputation. This class even now try to conceal their cases of this disease, and avoid for themselves and for their deranged friends all publicity, especially those places of healing on which public attention is concentrated, and desire to avail themselves of less conspicuous means of regaining their health. Some are very sensitive as to their disorder. They are conscious that they have difficulty in the head, and trouble in the mind, and fear that it may grow worse, and they are willing to confess so much. But they are unwilling to admit that they are insane, and are pained or irritated when they are supposed to be so; and even when the subject of insanity is mentioned in connexion with them, they are disturbed. The proposal to remove them to an asylum for the insane, known and recognised as such, confirms their fears that their dreaded enemy is believed by their friends to be upon them. Even after their entrance into the wards of the hospital, carrying the same conviction, some still rebel against the admission of their lunacy, and are disturbed and pained by their associates whom they know to be insane. They are offended with the strange manner and conversation, the excitements and depressions, the laughing and the weeping, the singular opinions and senseless jargons which are about them. They are consequently unreconciled to their position, unwilling to submit to the necessary requirements and restraints of the institution. They do not co-operate readily with the officers and attendants in their endeavours to heal them, nor lend the aid of self-discipline in removing their delusion. For those insane patients who can be managed under personal influences and in proper circumstances, there are some advantages and privileges which can be enjoyed in a higher degree in private and discreetly-managed houses than in public establishments. Insanity does not usually affect all the powers of human nature. It is rare that a patient is unsound in all his mental and emotional elements. Commonly only a part of them are disturbed, while the others are left in health. Some have only a single delusion, while on all other matters they think and talk rationally. Some have too much excitement, others too much depression. Some are excited only in a certain line of ideas, only in connexion with certain subjects, while in connexion with others they are calm. In some, certain appetites are morbidly active, and in others different appetites are wrong, while the rest are healthy. The moral affections have a similar variation of health and disease, of acquiescence and disturbance in the same patients.

"In all healing of disease, whether of body or mind, it is considered both philosophical and necessary, not only to interfere as little as possible with all the parts of the constitution that are sound, but to encourage and sustain them in carrying on their natural processes and discharging their healthy functions, and thereby obtain through them as much strength as possible for the constitution, and enable it to throw off the load that is imposed upon the others. Therefore a discreet physician or surgeon, in treating the disease of any organ or function, administers his remedies in such manner as not to disturb or impede the operations of the others. In treating disease of the lungs, he is careful not to impair digestion. In healing a local abscess, he cautiously sustains the nutritive powers, and thus he holds all the healthy functions as his allies, to aid in subduing the special disorder. On the same principle, the wise manager of the insane carefully analyzes the condition of his patients, and ascertains what elements are diseased and what are sound. Having determined this, he cautiously respects and avoids all interference with every power and faculty, every principle, opinion, emotion, taste, or desire, that is in good health, and applies his influence only to such as are not in good condition, and this he does in such a way as not to disturb the others. He therefore, so far as is consistent with the patient's recovery or best progress, applies no restraint, opposes no purpose, denies no indulgence, contradicts no opinions that are not disordered and do not minister to the disease. Thus he sustains as great an amount of healthy mental and moral constitution as possible, by means of which he hopes to overcome the disturbance in those which are diseased.

"Although most patients need the restraints which can be found only in public establishments, and cannot therefore be safely and properly treated elsewhere, yet there are some to whom all the peculiarities of such an institution are not necessary. Some need none of the restraints which the architectural arrangements of the building and the surrounding enclosures afford, nor the usual and necessary vigilance of attendants of the hospital, to prevent their doing harm to others or to themselves, or to retain them within the bounds appointed for them. On the contrary, the presence of these means of security is painful to some. There are a few trustworthy patients, some with and some without attendants. They can be allowed a wider range of motion, they can have freer walks, rides, and other means and opportunities of various exercise and change of scene, not only without detriment, but with advantage. Some are not offensive to ordinary and domestic social life, nor are they disturbed by its circumstances, occurrences, and interests. They can live in judicious families, eat at the table, and sit in the parlour with the household, and enjoy much of their company and friends. They may not always contribute to the enjoyment or the harmony, nor aid in the smooth flow of the domestic current; some do this very little, yet they derive great comfort from such relations to the ordinary family circle. Some can bear to be even more in the world, and engage to some extent in the general social life. They can visit and be visited with profit to themselves. Some can attend places of amusement, church, and other general gatherings, and receive no injury but rather benefit from this intercourse with the world. Of course such patients should be under the constant supervision of a suitable, discreet, and intelligent physician, who understands mental disorders and their origin, and who is willing to give himself, heart, soul, and mind, to this work. He must exercise an unremitting watchwe manager fulness over those entrusted to his care, noting all their variations of thought and feeling, of temper and propensity. He must arrange and control all the circumstances that surround them, and regulate all the influences that may bear upon them. The company, the conversation, the suggestions, the objects of interest, the scenery, their exercise and occupation at home and abroad, their diet, their sleeping, everything concerning them, must be under his unremitting watchfulness. These and all interferences and indulgences must be shaped, directed, and applied in each particular case, and at each particular occasion, according to the then present condition of the patient, and to the probable effect on his health, in the judgment of the physician. The same discretion and reliability, and a good degree of intelligence and fitness for his position, is necessary in the attendant. As he is to be the constant companion of the patient, he should resemble him as nearly as possible in character, education, and general culture, so that he may be an agreeable, not a wearisome associate, a pleasant and influential guide, not a mere servant to obey his commands, or yield to his caprices.

"The hospital is necessarily more inelastic than the private family. The rules are made to cover over the average of cases, but they must include the worst, so that nothing wrong may happen. They cannot be varied, either in the enactment or in the application, to suit the varieties of character or taste. But in the administration of a family there is no need of a written or printed code. The general laws of right and propriety are admitted, and such other directions may be given from time to time as may be needed for the insane inmates, as easily as such regulations may be made and altered in reference to a patient sick with any other malady. Hence each day's domestic administration may be made to suit exactly the condition of the lunatic at the time, and the cautions, restraints, and indulgences, varied as his good may require. As every influence that bears upon the patient may affect him for good or for evil, none should be allowed to reach him but such as are of themselves true, sane, and favourable. Not only the physician, his family, and the attendants, but all that come in contact with him—his associates, the visitors, the servants that wait upon him, the people whom he visits—should all be persons of well-balanced minds, and discreet bearing and habits; so that no insane ideas may be suggested, no wrong emotions excited, but every influence from without tend to keep his mind and feelings in a serene and cheerful state, and increase his power to think, feel, and act sanely.

"There is still a smaller class of patients, who need even less restraint and vigilant guardianship, but still must be separated from the familiar scenes of home and friends. It is not necessary for these to reside in the family of a physician, yet they need his supervision and guidance, and should therefore be in his neighbourhood, where he can know of their condition and movements, and visit them as often as they may need. They may be boarded in discreet families, and enjoy most of the common privileges of the household, and the ordinary attentions and comforts of domestic life. Being under proper medical supervision, all the healing influences of both physical and moral nature that they require may be secured for them, and their health re-established if recoverable, or they may be cared for and protected without suffering any needless privation of comfort.

"The views herein given refer only to that class of mild patients who are manageable in a private house, and who need not the efficient government and the restraints that are found in large institutions. The class referred to is not large, and some of them may be as well and as comfortably managed, and regain their health as certainly, as in an hospital. This of course does not include all the private institutions, for some of them are large, and have as many patients as the public establishments. I have only described one class, such as is most familiar to me.

"The proper function of private asylums or homes for the insane seems to be, not to compete with the public institutions in matters of cheapness; but to provide liberally for all the proper wants of their inmates, and charge all for material, time, attention, and responsibility, and receive a corresponding reward.

"Not to receive and treat the violent, the maniacal, the suicidal; but the mild, quiet, and manageable by personal influence.

"And principally to provide and offer to such patients as can properly enjoy and profit by them, an opportunity of using more of their faculties that are sane, a freer range of occupation and action, more of domestic and social life, more intercourse with the world, and a condition resembling more nearly that of their own homes than can be offered and enjoyed in the public hospitals."—(*American Journal of Insanity*, July, 1860.)

---

3.—*On the Management of the Insane in Belgium.* By Dr. J. Parigot.

The success or not of establishments instituted for the relief of the insane may be said chiefly to depend upon one principle, to wit, the ratio of approximation existing between the administrative and medical departments of the establishment. In proportion as these are in antagonism, we shall find the condition of the insane more or less unsatisfactory; in proportion as they approximate in object and in action, we shall find the state of the lunatic more or less ameliorated. The great aim of medicine is to make every asylum a hospital for the cure, not a prison for the detention, of the insane; and that asylum is the most perfect one which could rightly have inscribed in great letters above its gates, the legend borne aloft by Dr. Parigot,—"*ici l'on guérit pour en sortir au plus vite*:" Quick to cure, reluctant to detain.

In a report* made by Dr. Parigot to the Brussels Society of Medical and Natural Sciences, we have a critical sketch of the present condition of the establishments for the insane in Belgium. We shall not follow the author's observations in detail, because, whatever shortcomings or successes are at the present time to be noted in the different Belgian asylums, they, as in this country, may be referred to the principle we have already laid down. We shall confine ourselves, therefore, to Dr. Parigot's general conclusions as to the requisites necessary for the further amelioration of the condition of the insane in Belgium, and to his remarks on the lunatic colony at Gheel.

And of the latter first. There has been of late a growing belief in England that the success of Gheel, as a lunatic establishment, was not such as might have been anticipated from the seemingly admirable principles on which it is now founded. Dr. Parigot admits the justice of the belief, but at the same time he advances an explanation why Gheel has latterly deteriorated, which is not a little instructive. His statement is briefly this:—

Before the law of the 18th June, 1850, came into operation, Gheel was a species of *port franc* for madmen. The entrance was easy, but

* "Observations sur le Régime des Aliénés en Belgique apropos d'un livre de M. Ducpétiaux, intitulé: Notice sur les Etablissements d'Aliénés des Pays-Bas." Par le Dr. J. Parigot. Bruxelles. 1859.

the exit was difficult. The communal administration, as well as certain contractors, regarded Gheel as a warehouse for storing lunatics, and they sought here and there for the insane in regular trade fashion, extolling the advantages Gheel offered in an economical point of view. The lunatics transmitted there were regarded as so much goods, and commonly finished their days under the charge of those who received them. These were the peasants (the fosterers) who were charged with the charitable work of tending upon the unfortunates, and who obtained *this favour* by paying a higher rate for their commodities and lands. Since 1803 another, a third element, became operative in Gheel. The principal communes which had patients there sent to Gheel a representative, whose duty it was to overlook the lunatics who were lodged and clad by the commune he represented. Brussels elected Dr. Parigot to perform this function for its lunatics. At the time of these appointments, Dr. Parigot tells us that the administration of the colony was in the saddest confusion, and that the position of the lunatics was perhaps even more cruel than that of the negroes he had seen in South America. The only check to unbounded neglect rested in the individual kindness of the keepers, but this was sufficiently sound in character and abundant in amount to effect great improvements. When backed by the support and aid of the representatives of the different communes, Gheel improved visibly after the appointment of these representatives, and everything bid fair for a thorough reform. But a difficulty arose which put a stop to further progress, and under the influence of which Gheel is gradually deteriorating to its original state. The interest of the principal communes was solely that of their patients; the interest of the executive of Gheel was that of the commune in an economical point of view. The one looked at the patients from a medical and charitable point of view: the other from a commercial. When, therefore, the disturbing element of inspection by the different communes was introduced, and when, subsequently, the central government interfered, and by the appointment of a committee of direction and inspection sought to amend matters at Gheel, the council of that commune at once entered into fiercest opposition. It held that *its rights* (!) had been invaded, and so successfully has it maintained its opposition, that the interference of the central government has been rendered of none effect, and the communal council is now free from any serious control. Moreover, the inspectors of the different communes have been withdrawn, and unless some check be again interposed, Gheel will revert to its ancient state. Dr. Parigot suggests a "very simple remedy" to this state of things. He proposes:—

1. To confine the duties of the local committee, as well as of all the committees of the kingdom charged with that task, exclusively to inspection.

2. To appoint a director responsible to the minister, or his representative, as well for the legal duties concerning collocations, as for the material and financial administration of the establishment.

3. To appoint the physician-inspector responsible director of all that relates to the moral, hygienic, and sanitary state of the establishment. This comprehends necessarily the classing of the keepers, the allocation or removal of the insane. A special register which could be consulted by those whom it might concern, and which would show the reasons which govern the classification, and the ordinary mode of distribution, of the insane among the fosterers. This register would be open to protests.

4. To appoint an assistant physician-inspector, to aid in the infirmary, to keep the medical register, to perform autopsies, &c., and who would be librarian of the establishment, and would help the inspector with the annual report.

5. A new and single statute would be required. Experience shows that regulations by royal or ministerial decree fail practically.

6. The committee, the director, and physician-inspector, &c., should correspond directly with the Minister of Justice; the two employés, nevertheless, would do this only in exceptional cases, when, for the good of the service, it is found that the hierarchical way will not suffice.

With respect to the further improvement of lunacy administration in Belgium, the conclusions which Dr. Parigot has arrived at, and which were adopted by the Society of Medical and Natural Sciences, were as follows:—

1. It is essential to organize in Belgium, either in the universities or in the great asylums, that branch of medical instruction which treats of psychiatry, in order to furnish a medico-psychological clinique.

2. It is requisite to organize the medical service of the asylums by making the staff of physicians proportional to the number of patients, so that in the curative arrangements no physician shall have charge of more than fifty cases.

3. It is necessary that this service be in every way similar to that of ordinary hospitals, in which the curative methods employed by the heads of the staff can be controlled by the visitors.

4. The posts of clinical assistants, and even of assistant-physician, should be placed *au concours* among the young medical men leaving universities. While waiting for a vacancy the elected should be sent to certain foreign hospitals.

5. A medico-psychological clinical establishment, containing fifty beds, is alone to be recommended in provinces where there is not yet an asylum; this establishment ought to be situated at a little distance from the chief town.

6. The older closed asylums, or the free, as Gheel, should always have a medico-psychological clinique.

It would appear from this highly-interesting pamphlet of Dr. Parigot's, that there are at the present time fifty-one lunatic establishments of all kinds in Belgium; and that while the number of insane in 1853 amounted to 4054, since that time (within five years) it has increased to 4508.

---

### 4.—*Patronal Asylums.* By Dr. Mundy.

In a brilliant article published in the Brussels *Journal of Medicine* (August, 1860), Dr Mundy contends that Gheel is neither a colony nor an establishment for the insane (Gheel n'est pas une colonie, moins encore un établissement d'aliénés), and he suggests that in future Gheel should be designated as a Patronal asylum (*Asile patronale*). This term was originally proposed by Dr. Bulckens, in a letter to Dr. Mundy. "Gheel," wrote the former gentleman, "so judiciously entitled by M. Jules Duval, the *paradise, the kingdom of madmen, is still considered by many as a hell, a colony of wretches,* objects of traffic and of profitable farming.

"This has led me to seek for a more fitting and significant appellation for Gheel, and I propose to substitute for the term *lunatic colony* (colonie d'aliénés) the term *patronal asylum* (asile patronal d'aliénés à Gheel).

"[Patronal, qui appartient au patron.—Patronus—Advocatus. Patron se dit d'un homme sous la protection de qui l'on se met pour avoir de l'appui, et d'un homme dont on obtient le secours dans une affaire, dans une circonstance difficile. (*Dict. de l'Acad.*)]

"This denomination expresses exactly the character and charitable practice of Gheel, *which far from being a colony, is a place of surety, a family refuge* (refuge de famille), *where the insensate find among the inhabitants patrons who protect them.*

"On examining very attentively the mode of treatment to which the patients are submitted in this '*patronal asylum,*' we think that henceforth it may be termed the *patronal regimen* (régime patronal).

"For ourselves, the *patronal regimen* indicates the cares, the protection, the defence, the liberty, the equality, that the fosterer, *as patron,* accords to *his boarder,* who lives with him in full liberty, and shares his labours and his harvests. The fosterer, in receiving and assimilating the insensate with the members of his family, *delivers him from a state of neglect, of abjection, of brutishness, in which he had probably lived until then, and becomes rightfully, by so doing, his patron.*

"Such a regimen embraces in itself free-air, family-life, and work. In adapting, therefore, any proposition to this system, we ought in future to say, —*Patronal asylum for the insane* (asile patronal pour les aliénés), *patronal regimen of insanity* (régime patronal de la folie),—substituting these terms for those at present in use, to wit, *colony of lunatics; free-air and family-life treatment,* &c. (colonie d'aliénés ; traitement à l'air libre et la vie de famille, &c.)"

Dr. Bulckens promises presently to give to the world his observations and opinions upon the patronal regimen followed at Gheel; and Dr. Mundy announces that he is preparing for the press a work on phrenopathic medicine. This work, judging from the table of contents, will prove to be of considerable importance. We shall look for the promised publications with great interest.

---

5.—*On the Curability and Cost of Insanity.* By Dr. JOSEPH A. REED.

INSANITY should be regarded as symptomatic of disease of the brain, and should be treated with the same promptitude with which pneumonia, fevers, or other severe diseases, are met and subdued; and if thus met, the probabilities of recovery will approach very near to a certainty, but if neglected the disorder will fix itself permanently, the curable stage will rapidly pass away, and hope will have but little left to rest upon.

The following, taken from the Report of Dr. Butler, of the Hartford

Retreat, is so applicable, that we quote it entire :—" When common-sense views of insanity shall prevail—when this shall be treated like other diseases, with a fairness and decision corresponding to the gravity of the disease, and the importance of the organs implicated by it, the proportion of incurable cases in the community will be correspondingly diminished. I know of no disease which so imperatively demands that it be met on the part of friends with frankness and decision toward the sufferer, and with a reasonable confidence and patience toward those to whose skill and experience the sufferer is intrusted. It is a reasonable claim, the justice of which should never be overlooked, that one who is willing to accept the grave responsibility of treating a case of insanity, should ever find both his feelings and opinions treated with respect and deference."

The following, from a foreign periodical, is to the point :—" How is it that, in pestilence, fever, or any other scourge of the human race, the physician is sent for without disguise, and the case at once committed to a professional hand? But in the dread and mysterious mental disease, where, in the first stage, time lost is far more precious than jewels; where medical treatment is valuable almost in proportion as it is early; where the most unreserved confidence to the medical man is dictated by prudence, and the utmost candour of friends and relatives is essential to his forming a correct diagnosis; then a fatal repugnance often exists to making the necessary statements, and a childish irresolution in submitting to the appropriate remedies."

Of 100 patients in the Western Pennsylvania Hospital, at the date of the last report, 70 had been insane before admission for a longer period than six months, and were considered incurable. Of 332 admitted since 1855, 173 had been insane for periods varying from six months to 20 years, and of this number only 28 had recovered; the balance remain monuments of neglect—a burden to themselves and their friends, or the community, and the source of ceaseless care and anxiety. On whom, then, should rest the responsibility of perpetuating the bondage of this terrible disease, if not on those who, having charge of the helpless sufferer, neglected to give him the advantages of proper treatment in due season?

The Massachusetts Commission on Lunacy for 1854, report, " that it is reasonable to suppose that four-fifths of 840 who have never been in hospitals in that State, might have been restored with proper means. Without doubt, an equally large portion of those who were sent to a hospital, but not until their day of cure was past, might have been restored if they had been sent in time."

Dr. Earle, in the report of Bloomingdale Asylum, gives it as his opinion that one of the chief obstacles to a more general recovery of the patients admitted into public institutions, and one of the principal causes of the great accumulation of deranged people in the community, is the neglect of removing them to an asylum as soon as possible after the commencement of the disease.

Dr. Kirkbride has repeatedly expressed the opinon that insanity in its earliest stages is generally curable, and that every week it is left without treatment goes to diminish the prospect of restoration. Dr.

L. V. Bell expresses the following opinion:—" In regard to the curability of insanity, there can be no general rule better established than that this is directly in the ratio of the duration of the symptoms." Dr. Edward Jarvis, of Dorchester, says: " If insane persons are allowed to enjoy the means of healing in the early stages of their disorder, about 75 to 90 per cent. can be restored to health."

These opinions are not the result of a theoretical knowledge of insanity, but are founded on a long experience in the treatment of the insane, and are amply sustained by the statistics of all insane hospitals.

From the Reports of the New York State Asylum, we find that of 511 discharged restored, 421 had been insane for a period less than one year.

In the twenty-sixth Report of the Hartford Retreat, we find that of 226 recent cases, 186 recovered; while of 203 old cases, only twenty-five recovered.

The New Hampshire Asylum, in 1858, discharged thirty-one restored; of these, twenty-seven were recent cases.

In 1837 and 1838 the M'Lean Asylum, Boston, discharged 146 restored; of these, 117 were recent cases.

In 1858, the Southern Ohio Asylum discharged seventy-three restored; of these, sixty were insane less than one year.

The Mount Hope Asylum reports in 1855 and 1857 ninety-six recent cases under treatment, of whom fifty-two recovered; and of ninety old cases, only seven recovered.

The Massachusetts State Hospital at Worcester reports from 72 to 93 per cent. of recent cases, and only from 15 to 31 per cent. of old cases restored per year, during a period of twenty-four years.

The Columbus Asylum record shows that during twenty years 73 per cent. of recent and only 25 per cent. of old cases were restored each year.

The Edinburgh Royal Asylum reports 218 recovered, and of these, 174 were recent cases.

The Glasgow Royal Asylum reports in 1853 116 recoveries; of these, ninety-one were recent cases.

Of 119 discharged from this hospital recovered, 101 were recent cases, and were under treatment for periods varying from one to twelve months. From a table prepared by Dr. Jarvis, of Massachusetts, embracing 4800 cases, we find the average time required for their recovery, under hospital treatment, was six months and sixteen days. In contrast with this, the duration of life of the uncured insane should cause every one in charge of recent cases to act at once in their behalf. From a table prepared by the actuary of the Albion Life Assurance Company, London, we learn that the average length of life of persons incurably insane, if attacked at twenty years of age, is twenty-one years; if attacked at thirty, it will be twenty years; if attacked at forty years of age, the probabilities are that the patient will live seventeen years.

There can be no question, then, we presume, about the curability of recent cases, and the necessity and humanity of subjecting them, at the earliest possible moment, to proper remedial measures; and the only doubt that can exist, is in regard to the expense of their treatment, or their support through a lifetime of lunacy. On this point we again refer to the records of other institutions.

Dr. Kirkbride, in his report for 1842, says: "By referring to the register of this institution, I find that the actual average cost of supporting the first twenty successive cases that were discharged cured, from the time of their admission, was only $52·50, while in the first twenty incurable cases that were received in the house, at the same rate of expense, from the time of the commencement of the disease till 1841, the average cost of each of their friends was $3,045."

In the Massachusetts State Hospital, up to 1843, twenty-five old cases had cost the sum of $54,157, while the same number of recent cases, until restored, had cost $1,461·30.

In the Ohio Lunatic Asylum, in 1842, twenty-five old cases had cost $35,464, while twenty-five recent cases, until recovered, had cost $1.608.

In the Maine Lunatic Hospital, in 1842, twelve old cases had cost $25,300, while the same number of recent cases had cost only $426.

In the hospital at Staunton, Virginia, twenty old cases had cost $41,633, and the whole expense of twenty recent cases, until restored, was only $1,265.

Certainly no one should hesitate in deciding between the expense of a few months' treatment, or that of a lifetime of insanity. Humanity and economy unite in their appeal for timely and judicious care of the insane.—*Report of the Managers of the Western Pennsylvanian Hospital for* 1859.

---

6.—*On Pellagra in Italy, and more particularly in the Lunatic Asylums of that Country.* By Dr. E. Billod.

Dr. E. Billod was commissioned, in January, 1859, by his Excellency the Minister of the Interior, to visit Italy, and report upon the pellagra, of that country in its relations with mental alienation. In the report which, in accordance with his instructions, he has recently presented to the Minister, he records not only the results of his researches in Italy in 1859, but also the results of previous researches which he had made in that country in 1846. The object of Dr. Billod's researches was a comparative study of the true pellagra, considered as a type, and of the affection incident to mental alienation admitted as a variety; to seek the degree of analogy existing between these two morbid species, and to decide, if possible, upon their identity of character.

The results of Dr. Billod's researches, and the conclusions that he has formed, are thus summed up:—

1. Pellagra is endemic, in different degrees, in the provinces of Perugia, Urbino, and Pesaro, in the States of the Church; in part of Tuscany (Tuscan Romagna and Mugello), in the Romagna, in Emilia, in the Milanais, and in part of Piedmont; and other parts of Italy seem to have, in this respect, a certain immunity.

2. In the countries where pellagra is endemic, it constitutes one of the most frequent causes of mental alienation among individuals admitted into lunatic asylums.

3. Mental alienation shows itself most frequently in the latter periods of pellagra, and most commonly assumes the melancholic form; but it is also observed at the commencement and in a maniacal form.

4. The disposition to commit suicide does not perhaps accompany the mental alienation of the pellagra-stricken so often as is imagined; and death by submersion is not commonly, as has been asserted, the form of death which is commonly chosen by the insane pellagra-stricken who manifest a suicidal tendency.

5. The opinion advanced by Dr. Billod that the cachexy which he has described as being peculiar to the insane, is of a pellagrous character, is confirmed by the observations made by him in many establishments, and particularly in those of Florence, Astino, and Turin.

6. Alimentation by maize, with or without modification by *verdet*, according to the general opinion of the Italian physicians, so competent in a question to decide which they are not reduced, as the majority of French physicians, to views purely theoretical, is one of the principal causes of pellagra, but *it is far from being the sole and exclusive one.*

7. The cause of pellagra is, according to the same physicians, complex and variable; that is to say, it results from a combination of many hygienic conditions, of which the use of maize forms but one.

8. Softening of the spinal cord, which Dr. Billod had noted as the most ordinary *post-mortem* appearance of the peculiar cachexy of the insane, especially in the pellagrous form, is also observed in patients who have suffered from true pellagra, as was demonstrated by Dr. Brierre de Boismont in four autopsies made by him at the Milan Hospital. The pathological change is not, however, peculiar to pellagra, for it is observed occasionally in various conditions of mental alienation, more or less independent of pellagra.

---

7.—*On Microcephalus considered in its relations with the Characteristics of the Human Race.* By Dr. P. Gratiolet. (*Read before the Société d'Anthropologie de Paris*).

I propose to communicate to the society some observations on microcephalus, dwelling on certain characteristics supplied by the study of arrests of development exclusively proper to the human group.

The microcephalics which I have studied belong to the category of dwarfs, often very elegant in their forms, which have of late been submitted to the public curiosity perhaps somewhat too freely. As they have nothing at first sight monstrous in their appearance, they have been sometimes put forward as specimens of certain pigmy races hitherto completely unknown. Thus five or six years ago there were exhibited in London and Paris, under the name of Aztecs, from the

United States, some small microcephalous dwarfs, evidently the issue of a cross between Negroes and Indians; for their curved nose, their tint at once copper-coloured and fuliginous, and finally, their crisped hair, revealed a double relationship with the American race and with some black race. Microcephalics, although preserving all the principal characteristics of their race, present, in some respects, a common physiognomy. Their figure is degagé and well proportioned, but their forms are not those of puberty; they recall that of a child of ten or eleven years. Their face is very prominent; their eyes and teeth are relatively enormous; the forehead is extremely retreating, whilst the occiput is, on the contrary, globulous. Be this as it may, however, the skull, which is singularly reduced in the cerebral, would appear deformed if it were not disguised by a covering of hair much thicker than usual, the mass of hair originally designed for a normal head being concentrated on a smaller space.

These little creatures are all, without exception, of extreme vivacity. They move, according to the simultaneous expression of all observers, with the lightness of a bird. This perfect co-ordination of their movements is astonishing if compared with the feebleness of their intellect. Generally very gay, capable of affectionate sentiment, but excessively capricious, they appear to be entirely deprived of the faculty of attention, and completely personify the idea which the Latins attached to the word *fatuus*. Several of them speak a truly articulate language, not rich, it is true, but really human in all its characteristics.

Thanks to the kindness of MM. Baillarger and Giraldès, I had a fortunate opportunity of studying the brain of three of these singular beings. One of them belonged to a Negro race; the other two were white, and were born in France.

The skulls of these three dwarfs were very small, a little less than that of the chimpanzee or the ourang. This excessive reduction was confined to the superior portion of the cranium—its base being but slightly ossified. In these three subjects the basi-occipital was separated by a cartilaginous disc from the basi-sphenoid, although one of the children was fourteen years of age. These bones were themselves almost entirely cartilaginous, although at the same time sufficiently voluminous. The petrous portion of the temporal and the ethmoid, far from having undergone any reduction, appeared to have acquired a greater development than in the normal state.

Whilst on the one hand the ossification of the base of the skull was imperfect, that of the vault was extremely advanced. The frontal, parietal, and occipital bones were dense and very thick. All the sutures which persisted were very simple, and but slightly undulated; the medio-frontal was absolutely effaced; the sagittal had left an apparent trace, but its obliteration was complete, and the transverse was beginning to disappear. A single suture then persisted; that is to say, the lambdoidal, which again was very slightly complicated.

Altogether, the skull-cap presented a very singular form. Its vault was not an elliptic arch, like that of young children, but a somewhat pointed ogee. Besides its general diminution, the cerebral fossa presented an excessive reduction in the frontal and epactal regions.

The parietal also, besides being much reduced, encroached evidently on the other two.

Regard being had to the smallness of the skull, the cerebellar fossa was of enormous dimensions. It projected behind and at the sides beyond the posterior extremity of the cerebral fossa, which was much restricted. It was much dilated above, terminated conically, and presented, as a whole, that form of a funnel, which M. Retzius has pointed out as peculiar to the occiput of the young fœtus.

The facts just indicated had had a very curious influence on the development of the different parts of the face. The superior arcs of the cranial vertebra being in some degree atrophied, their inferior arcs had acquired an excessive development. The pterygoid bones, the bones of the palate, and the intermaxillaries, had, in developing themselves, drawn into their movement the upper maxillaries, and the entire upper jaw offered a marked projection; the lower jaw, on the contrary, independent, as we all know, of the vertebral series, had retained its normal form and proportions; the result was inevitable. The development of the two jaws taking place unequally, they no longer corresponded in front, and the upper incisives no longer met the lower.

This deformity often accompanies foolishness, and then it gives the face an expression of characteristic silliness; it is so closely connected with the diminution of the frontal arc, that it is met with in the heads of Caribs and Aymaras, who have a custom of flattening or deforming the forehead. The skulls in the collection of the museum show this. We might also refer to those figured by Morton in his fine work *Crania Americana*; and looking at the jaws of *Botocudo*, which he has represented in his fifteenth plate, one might imagine that this very short head had been artificially deformed. However this may be, this deformity appears to be an inevitable consequence of microcephalus. It existed in the observation of microcephalus published by Gall. The three microcephalics which I have studied also exhibited it; it was most highly marked in the case of the pretended Aztecs, as any one may convince himself by an examination of the very accurate portraits made by M. Barwell in London, by the desire of M. H. de Saussure; and it was more especially evident in the boy, who was more microcephalous than the girl; it was also exhibited in the case of the pretended Earthmen. It gave, to the advantage of those poor little creatures, the birdlike physiognomy which is so striking in the drawings of M. Barwell.

This imperfect and monstrous proclivity of the face differs from the natural proclivity which constitutes prognathism; in the case of the Makuos, a race of Southern Africa, whose prominent muzzle immediately recalls the physiognomy of the gorillas and cynocephalous papions, the lower jaw invariably corresponds perfectly with the upper, which proves that in this case the proclivity is normal, and does not depend upon an accidental degradation. I think that this fact furnishes an additional argument to the partisans of the plurality of species in the human genus.

The study of the brain of microcephalics has provided me with other elements, by the aid of which the absolute distinction of man is evidently and anatomically proved. In comparing attentively the brain of monkeys with that of men, I have found that, in adult age, the arrangement of cerebral folds is the same in one group as in the other; and were we to stop here, there would not be sufficient ground for separating man from animals in general; but the study of development calls for an absolute distinction. In fact, the temporo-sphenoidal convolutions appear first in the brain of monkeys, and are completed by the frontal lobe, whilst precisely the inverse order takes place in man: the frontal convolutions appear first, the temporo-sphenoidal show themselves last; thus the same series is repeated in the one case from *a* to *ω*, in the other from *ω* to *a*. From this fact, rigorously verified, a necessary consequence follows: no arrest in the progress of development could possibly render the human brain more similar to that of monkeys than it is at the adult age: far from that, it would differ the more the less it were developed. This consequence is completely borne out in the brain of the microcephalic; in the first instance, it might be taken for the brain of some new and unknown monkey, but the slightest attention would suffice to enable us to avoid this error. In a monkey the parallel fissure would be long and deep; the sphenoidal lobe would be charged with complicated incisures. In a microcephalic, on the contrary, the parallel fissure is always imperfect, and sometimes absent, and the sphenoidal lobe is almost perfectly smooth.

This is not all; in the case of microcephalics, the second fold of the passage between the parietal and occipital lobes is always superficial, which is a characteristic absolutely proper to man. In the brain of pitheca, on the contrary, this fold is constantly hidden under the operculum of the occipital lobe. Thus, in the midst of their degradation, the brain of microcephalics presents human characteristics. Though often less voluminous and less involved than that of the ourang-outang, or the chimpanzee, it does not become like theirs. The microcephalic, however reduced, is not a beast; he is a dwarfed man.

I have examined whether microcephalus preceded or not the birth. I find incontestable evidence that it does. In one of the microcephalics that I studied, the general form of the brain and the fissure of Sylvius showed that the deformity was at least contemporaneous with the fifth month. It seems probable that this state depends upon some initial cause. Under the influence of a primordial astheniogeny, forms are produced which differ from all normal conditions; but in a normal new-born infant, the system of cerebral folds is complete in all its parts.* If microcephalus appeared after birth, all the folds would remain, and the volume only of the brain would be diminished; but it is not thus. The movement has languished from its origin, its curve is shortened, it has finished prematurely, and far short of its normal end.

Perhaps I ought here to direct attention to the enormous development of the cerebellum, for these beings never attain puberty. This fact is little favourable to the theory of Gall, but is much more so to

* It is the same with all animals that are born with their eyes open; in the case of those born with closed eyes, the convolutions are only perfected at the moment when the eyelids separate.

that of M. Flourens. The normal microcephalics move about with perfect rapidity, ease, and harmony. A very large relative development of the medulla spinalis and oblongata no doubt contributes to their agility.

Thus the reduction takes place more especially, and almost exclusively, in the cerebral hemispheres. The external organs of sense are large and well developed. The nerves leading to them exhibit a development surpassing the dimensions of the normal state.

Having attempted to demonstrate that the microcephalics preserve the material or zoological characters of man, I would remark that they also retain man's proper intellectual aptitudes. Most of them have an intelligible language, poor, it is true, but articulate and abstract. Their brain, inferior in appearance to that of an ourang or a gorilla, is nevertheless that of a *speaking mind.* This innate, and so to speak, ineffaceable virtuality, is certainly man's noblest and most brilliant characteristic. This strikes us especially within sight of the partial attenuation and ruin of the intellectual organs. Disease and astheniogeny may dwarf man, but they do not make him a monkey.

These microcephalics, deprived of convolutions, are all very small dwarfs. This recalls to mind the relation supposed to have been discovered some years ago between the development of convolutions and that of figure. It is true that all large animals possess cerebral convolutions, and that a great number of small animals do not. But this relation appears to me to have been ill appreciated. It is, on the contrary, the development of convolutions which announces that of figure, always preceding it, *not alone in the individual, but in each zoological group as a whole.* Thus, in the natural groups which contain gigantic animals, the smallest species have convolutions, whatever in other respects the exiguity of their figure. Such are the weasel amongst the carnivorous plantigrades or palmigrades, the *Antelope hemprichiana* (*Ehr.*) and the *Spinigera* (*Temm.*) amongst the ruminants.

Among human races, the Bosjemann has convolutions very little complicated. The frontal lobe especially presents a degree of simplicity which is never met with in white races, except in some cases of congenital idiocy. This is a race whose figure is very small; at the same time the Bosjemanns are neither microcephalous nor idiots. This sufficiency of an imperfect cerebral form proves that it is normal, and in some sort specific; and that, if the Bosjemanns are men, anthropologically inferior, they cannot with any reason be considered as degraded. Their race is fruitful. This is proved by its duration in the midst of the destructive causes which necessarily surround it. It is therefore not degenerate; for all modern observations agree in demonstrating that all degeneracy has a fatal termination in proximate sterility.

I think I may conclude, from the preceding observations, that man is absolutely distinguished from the highest orders of animals, no less by his organization than by his intelligence. He alone has an essential language, by reason of the faculty of abstraction, which is proper to him alone. Animals, the ourang, and the chimpanzee, without doubt, have ideas of external objects; their incontestable memory proves it, but the idea is essentially tied to that of its object. Man alone is capable of the idea of an idea; so that the intelligence of a beast is as a simple number, but that of man is as a power, the exponent of which is higher or lower according to the degree of perfection of individuals and of races.—(*Journal de la Physiologie de l'Homme et des Animaux. Janvier*, 1860.)

---

### GHEEL.—LETTER FROM DR. WILLERS JESSEN.

